# Emotional–Social Intelligence Profiles and Adaptive Professional Functioning Among Teachers: A Person-Centered Analysis of School Climate Perception and Conflict Handling

**DOI:** 10.3390/jintelligence14080184

**Published:** 2026-08-10

**Authors:** László Balázs

**Affiliations:** Department of Organizational Development and Communication Science, Institute of Social Sciences, University of Dunaújváros, 2400 Dunaújváros, Hungary; balazsl@uniduna.hu

**Keywords:** emotional–social intelligence, Bar-On EQ-i, latent profile analysis, person-centered approach, teachers, school organizational climate, conflict management, three-step BCH

## Abstract

Research exploring the relationship between emotional intelligence and workplace outcomes is predominantly variable-centered and treats the dimensions of emotional–social competencies as separate from one another, thereby losing sight of the intra-individual interplay of these competencies and the potential heterogeneity of their effects. This study examines teachers’ emotional–social intelligence using a person-centered approach. Using a sample of 587 Hungarian teachers from 26 public education institutions, we conducted latent profile analysis based on Bar-On’s five composites and then used unbiased three-step procedures (BCH; categorical BCH) to examine the antecedents of profile membership, as well as perceptions of school organizational climate and dominant conflict management style. Four distinct profiles emerged, primarily differing at the global level—a uniformly low profile (12%), a medium-low profile (25%), a dominant, balanced medium-high profile (53%), and a uniformly high profile (9%). Participants with higher profiles perceived the school’s organizational climate as more supportive (η^2^ = 0.07) and exhibited more adaptive conflict management patterns (less avoidance, more problem-solving; χ^2^(12) = 37.24, *p* < .001). A compositional (log-ratio) reanalysis of the complete conflict-mode profiles, reported as the primary conflict analysis, showed that the relative weight of problem-solving rises across profiles and plateaus between the modal and the highest profile, that avoidance declines with profile elevation, and that an apparent surplus of accommodating in the highest profile was an artifact of dominant-style coding rather than an indication of a broader repertoire. At the same time, profile membership was essentially independent of job position, career stage, and gender, and the relationships with outcomes were of modest to moderate strength. The results extend person-centered, profile-based intelligence research to the emotional–social domain and identify promising directions for research on teacher professional development.

## 1. Introduction

Emotional intelligence is a key—yet still controversial—construct in the psychology of individual differences. Its central premise is that differences in the perception, understanding, regulation, and social application of emotions are substantially related to how effectively an individual adapts to the emotional demands of their personal, social, and workplace environments (Mayer & Salovey, 1997; Bar-On, 2006; Mayer et al., 2016). This functional–adaptive perspective is particularly relevant in professions that involve sustained emotional labor, intense social interactions, and a constant need for self-regulation. The teaching profession is a prime example in this regard: teachers’ daily work takes place within emotionally charged relationships with students, parents, colleagues, and administrators; therefore, emotional and social competencies are not merely individual traits but potential resources for professional functioning and organizational adaptation (Brackett et al., 2010; Jennings & Greenberg, 2009).

The relationship between emotional intelligence and workplace and educational outcomes is supported by an extensive body of empirical literature. According to previous studies and meta-analytic findings, EI is associated with performance, well-being, job satisfaction, lower levels of burnout, and more constructive social functioning, although the strength of these associations also depends on how the construct is operationalized and the type of measurement instrument used (Joseph & Newman, 2010; O’Boyle et al., 2011; Mérida-López & Extremera, 2017). In educational research, there is particularly strong evidence indicating that emotional competencies play a protective role against burnout, stress, and difficulties adapting to one’s career (Fiorilli et al., 2019; Puertas Molero et al., 2019; D’Amico et al., 2020; Lucas-Mangas et al., 2022). However, these studies are predominantly variable-centered: they treat the individual dimensions of EI as separate predictors and estimate their average, population-level associations with various outcomes.

This approach has revealed important correlations but is of limited use in capturing how emotional–social competencies are organized within a single individual. Teachers may differ not only in terms of having higher or lower overall emotional–social intelligence, but also in the configuration of their competencies: for example, someone may be strong in interpersonal skills while having less developed stress management or adaptive skills. Variable-centered models necessarily obscure such intra-individual patterns, as they estimate the additive and person-independent effects of the dimensions. Thus, they do little to answer the question of for whom, under what competency patterns, and under what organizational conditions emotional–social intelligence becomes an adaptive resource (Howard & Hoffman, 2018; A. J. S. Morin et al., 2018; Spurk et al., 2020).

The person-centered approach offers a solution to this very shortcoming. Latent profile analysis identifies not the isolated effects of individual variables, but rather patterns of competencies that co-occur within individuals, thereby making it possible to examine how teachers’ emotional and social intelligence is organized into subgroups. Previous person-centered research on EI and emotional labor conducted on non-teacher samples suggests that profiles of emotional functioning may differ not only at the global level but, in certain cases, also in their structure, and that these profiles may be linked to various well-being, performance, or social outcomes (Gabriel et al., 2015; Toyama & Mauno, 2016; Pirsoul et al., 2023). However, such a profile-based examination of emotional–social intelligence, linked to organizational outcomes in a sample of educators, is still lacking.

The present study aims to address this research gap. Using a sample of 587 Hungarian teachers from 26 public education institutions, we examine which latent profiles can be distinguished based on the five composites of Bar-On’s emotional–social intelligence—intrapersonal, interpersonal, stress management, adaptive, and general affective skills. We interpret the profiles not in isolation but within their professional and organizational contexts: on the one hand, we examine whether profile membership is related to job position, career stage, and gender; on the other hand, we analyze whether the individual profiles differ in terms of the perceived school organizational climate and the dominant conflict management style. We estimate the relationship between the profiles and the external variables using three-step, unbiased procedures that account for the uncertainty of class assignment and thus offer a more reliable alternative to the traditional “classify-then-analyze” approach (Vermunt, 2010; Asparouhov & Muthén, 2014; Bakk & Vermunt, 2016).

This study offers three main contributions. First, it complements the variable-centered approach in emotional–social intelligence research with a person-centered analysis, thereby examining EI not as a set of isolated dimensions but as a pattern of competencies within an individual. Second, it extends profile-based intelligence research to a population that has been little studied to date but is particularly relevant from both theoretical and practical perspectives: teachers. Third, it links emotional–social profiles to two organizationally significant outcomes—perceptions of the school’s organizational climate and conflict management style—thereby nuancing the view that the role of EI can be interpreted merely as a linear, global resource level. This approach not only contributes to a better understanding of the structure of emotional–social intelligence but can also provide more nuanced guidance for teacher professional development and school leadership development. More broadly, the study integrates the professional-demographic connections and organizational correlates of emotional–social intelligence within a single person-centered framework, and thus addresses the question of how intra-individual patterns of these competencies co-occur with perceived indicators of adaptive professional functioning over the course of a long career spanning multiple career stages.

## 2. Theoretical Background

The theoretical elaboration of the central problem outlined in the introduction follows the line of reasoning below. First, we clarify what we mean by emotional intelligence, and—through an evaluative review of competing conceptualizations—justify why Bar-On’s model of emotional–social intelligence and its five composites provide the appropriate framework for the present study, while also demonstrating that the structure of this model itself renders the configuration question theoretically meaningful (Section 2.1). We then present the person-centered approach as an appropriate operationalization of this question and outline the burden of proof it must bear (Section 2.2). We then identify the teaching profession as the population and context in which this question becomes particularly interesting (Section 2.3); finally, we provide a theoretical basis for why emotional–social intelligence profiles are expected to vary across school organizational climate and conflict management (Section 2.4).

### 2.1. Conceptualizations of Emotional Intelligence and the Bar-On Model

Conceptualizations of emotional intelligence can be divided into three traditions that remain relevant today; it is useful to distinguish them not merely by their names but according to their measurement philosophies (Ashkanasy & Daus, 2005; O’Boyle et al., 2011). The ability-based model defines emotional intelligence as the capacity to process emotional information—a construct similar to cognitive intelligence that can be measured by performance tests; it posits four hierarchically organized branches—the perception of emotions, the use of emotions to aid thinking, the understanding of emotions, and the regulation of emotions—and operates on the principle of maximum performance, with correct and incorrect responses (Mayer & Salovey, 1997; Mayer et al., 2016). The trait-based approach, by contrast, focuses on emotional self-perceptions and dispositions, and locates the construct as a lower-order trait within the personality domain, defined as “emotional self-efficacy” as measured by self-reports (Petrides & Furnham, 2001; Petrides et al., 2007). Finally, mixed models integrate abilities, dispositions, and motivational facilitators into a single, broad construct (Bar-On, 2006; Boyatzis et al., 2000; Goleman, 1995); the most fully developed representative of this tradition is Bar-On’s (2006) Emotional–Social Intelligence (ESI) model, which organizes interrelated emotional and social competencies into five main composites: intrapersonal, interpersonal, stress management, adaptive, and general affective skills.

Among the mixed models, we selected the Bar-On framework on three grounds. First, its five-composite structure provides multidimensional indicators of comparable breadth, which is a precondition of profile analysis; narrower instruments, such as the four-dimensional WLEIS (Wong & Law, 2002), offer fewer and more homogeneous indicators and thus a smaller configurational space for a person-centered model. Second, in contrast to competence frameworks building on Goleman’s model, the EQ-i is a psychometrically documented self-report instrument with an established subscale structure. Third, a validated Hungarian adaptation with instrument-specific scoring was available, which is essential for the comparability of composite scores.

These three traditions are not equivalent, and the choice between them has significant implications. The ability-based approach is theoretically the clearest—it treats EI as a true form of intelligence—but its measurement tool (the MSCEIT) is not ideal for the present study for three reasons: performance-based scoring relies on a consensus or expert rubric, the validity of which is disputed; its predictive validity for workplace and well-being outcomes is, according to meta-analyses, weaker and less consistent than that of mixed- and trait-based measures (Joseph & Newman, 2010; O’Boyle et al., 2011); and a long, complexly scored performance test is practically unfeasible in a field study involving 587 participants across 26 institutions. The trait-based approach is self-report-based and well-suited for field use, but it places the construct explicitly at the lower level of personality, which pushes the language of competence and intelligence into the background.

We did not choose the mixed/ESI model because it is free from criticism—quite the contrary. The Bar-On model has been justifiably criticized for its conceptual breadth, for conflating ability and disposition, and for its significant overlap with the basic dimensions of personality (particularly neuroticism and extraversion) (Conte, 2005; Joseph & Newman, 2010; Matthews et al., 2002). Our choice, made with full awareness of these limitations, was based on four considerations. First, regarding the issue of configuration, the appropriate level of aggregation is crucial: the Bar-On model’s five broad, theoretically well-organized composites represent precisely the level of resolution at which the intra-individual configuration of competencies can be meaningfully described and substantively interpreted—as opposed to a single global performance score, which would obscure the profile structure from the outset. Second, the Bar-On model is explicitly formulated within an adaptive and developmental framework: the ESI is a system of developable competencies, which aligns directly with the practical and developmental relevance of our study and is consistent with the intervention evidence demonstrating that teachers’ emotional competencies and self-efficacy can be developed (González-Ros et al., 2026). Third, it has a validated Hungarian version of the EQ (Kun et al., 2012; Kun, 2011; Balázs, 2020), and has proven to be a valid, informative measurement tool in the teacher population, thereby fulfilling the population-specific condition of measurement validity. Fourth, mixed measures—precisely because of their overlap with personality and motivation—demonstrate incremental predictive validity for workplace outcomes beyond cognitive ability and basic personality traits (O’Boyle et al., 2011; Joseph et al., 2015)—and it is precisely these organizational adaptation outcomes that we are examining. At the same time, we do not ignore the criticisms: since mixed EI measures self-perceived competence rather than peak performance, we consistently interpret the profiles as emotional–social competence self-perceptions rather than objective abilities, and we treat personality-related antecedents and self-report outcomes with the appropriate caution (see Section 2.4 and Section 5).

From the perspective of the present study, however, it is not the number of dimensions in and of itself that is decisive, but rather the structure of the relationships among them. The five ESI composites are both correlated and distinct: they are linked by moderate-to-strong correlations—suggesting the existence of a common, general emotional–social capacity—yet they possess independent theoretical content. This duality is clearly demonstrated by the bifactorial modeling of emotional abilities: the structure simultaneously includes a strong general factor and independently interpretable, broad ability factors (Simonet et al., 2021). Emotional intelligence is thus hierarchical and multidimensional, similar to other areas of intelligence. This structure has direct implications for the profiles. If the ESI were underpinned by a single general factor alone, individuals would essentially be located on a single continuum of “more or less emotional capacity,” and the profiles would differ merely in elevation. If, however, the broad dimensions also carry genuine, partially independent variance—and the bifactor evidence suggests this is the case—then it is theoretically expected that there are individuals whose shape of competencies differs. Differences in the elevation and shape of the profiles are thus not mutually exclusive but equally well-founded expectations. Variable-centered research practice breaks down precisely this structure: when we treat the five dimensions as separate regression predictors, we implicitly assume that their effects are additive and independent of the individual—that is, we discard precisely the configuration information that the construct’s own, co-occurring-yet-distinctive structure renders relevant.

### 2.2. The Person-Centered Approach and the Burden of Proof

The person-centered approach provides a theoretically adequate response to this proposition (Section 2.1). It is rooted in the holistic-interactionist view of personality, according to which the individual is an organized, dynamic whole in which psychological variables do not operate in isolation but as interacting patterns; it follows that the appropriate unit of analysis is not the variable, but the person (Bergman & Magnusson, 1997; von Eye & Bogat, 2006). The contemporary methodological counterpart to this approach is latent profile analysis (LPA), which, as a model-based clustering method, identifies unobserved subgroups hidden within a population based on continuous indicators (Bauer, 2022; Spurk et al., 2020). The person-centered logic directly operationalizes the question “for whom”: it does not estimate how, on average, a dimension is related to an outcome, but rather what types of people exist and how these types differ (Howard & Hoffman, 2018; Meyer & Morin, 2016; A. J. S. Morin et al., 2018). The two paradigms are not rivals but complementary: the variable-centered model describes average, linear associations, while the person-centered model reveals configurational information that goes beyond this (Marsh et al., 2009).

That this approach is fruitful in the field of emotions and emotional intelligence is demonstrated by a body of literature that is now well-established but has been scarcely utilized in educational research. In organizational psychology, Gabriel et al. (2015), by examining the two strategies of emotional labor (surface and deep regulation) not as additive predictors but as intra-individual combinations, identified five emotional labor profiles—each linked to different antecedents and associated in distinct ways with emotional exhaustion, job satisfaction, and perceived inauthenticity—demonstrating that it is the combination of strategies, not merely their level, that is decisive. Toyama and Mauno (2016) identified six trait-EI profiles among nurses and—crucially for the present study—demonstrated that outcomes are influenced not only by the global level but also by the disproportion of the subscales: an “imbalanced” profile characterized by high interpersonal but low situational EI showed poorer well-being and performance than would be expected based on the absolute levels alone—this is precisely the kind of shape effect that remains invisible to a variable-centered approach. Pirsoul et al. (2023), using a sample of more than 1500 participants, identified four EI profiles that differed in both level and structure, which predicted career outcomes differently by gender; C. L. Thomas and Heath (2022), as well as Haag et al. (2025), used similar logic to link ability- and trait-based EI profiles to academic, well-being, health, and decision-making outcomes. In the field of education, Rentzios et al. (2025) used a person-centered analysis to identify both coherent and dissonant emotional-motivational student profiles and linked them to academic performance. These precedents offer a twofold lesson: on the one hand, they establish a well-founded expectation that emotional–social intelligence should have distinguishable profiles—varying in level and form—even among educators; on the other hand, they provide a model for correctly linking these profiles to distal outcomes. However, this approach is still lacking when it comes to teacher samples and organizational outcomes.

However, the person-centered approach also places a clear burden of proof on the researcher, which must not be overlooked. In practice, LPA often yields a solution in which the profiles differ primarily in elevation—at the global level—with barely distinguishable shapes. The methodological framework developed to analyze similarities between profiles distinguishes four dimensions: configurational, structural, dispersional, and distributional similarity (A. J. S. Morin et al., 2016). A structure that is predominantly elevation-based, organized “from high to low,” can easily be confused methodologically with a single continuous latent dimension, raising the legitimate objection: if the groups are merely intersections of a level continuum, what does the profile approach add to variable-centered analysis? There are two ways to substantively respond to this criticism: either by demonstrating structural differences among the profiles, or by showing that the profiles are related to external variables—antecedents and outcomes—in ways that mere elevation does not predict. This study adopts this dual standard: it not only identifies the profiles but also tests their antecedents and organizational correlates using unbiased three-step procedures that account for classification uncertainty, and it also conducts a direct comparison with variable-centered models (see Section 3.3 and Section 4.7).

### 2.3. Emotional and Social Intelligence in the Teaching Profession

The issue of configuration and heterogeneity is particularly important in the teaching profession, which is a prototypical arena for sustained emotional labor. The concept of emotional labor captures the requirement that workers regulate and express their emotions in accordance with organizational expectations (Grandey, 2000; Hochschild, 1983); in the teaching profession, this requirement is sustained and multifaceted, as interactions with students, parents, and colleagues demand continuous emotional regulation. Teachers’ emotional and social competencies are therefore not neutral individual traits, but rather central resources for the quality of teaching and the social-emotional environment in the classroom (Jennings & Greenberg, 2009). Empirically, individual differences in this area are consistently associated with the three classic components of burnout (Maslach et al., 2001) and teacher well-being: the protective role of emotional competencies is confirmed by both individual studies and systematic reviews (Brackett et al., 2010; Mérida-López & Extremera, 2017; Fiorilli et al., 2019; Puertas Molero et al., 2019; Xue et al., 2026; Cervellione et al., 2025). D’Amico et al. (2020), for example, demonstrated in a study of 238 Italian teachers that perceived emotional intelligence is positively associated with workplace engagement and satisfaction and negatively associated with burnout, which supports the protective role of EI. Studies examining the mediating roles of teacher well-being and psychological resources also indicate a similar pattern (Lucas-Mangas et al., 2022; Suárez Martel & Martín Santana, 2021).

Two additional considerations make the teaching profession a particularly suitable field of study. First—and this underpins the practical relevance of the profiles—emotional and social competencies can be developed: intervention research conducted among teachers confirms that emotional awareness and self-efficacy can be enhanced through targeted programs (González-Ros et al., 2026). If ESI is malleable, then the identification of profiles is not merely descriptive but also has implications for professional development and intervention: it enables differentiated, profile-tailored continuing education. On the other hand, the teaching community is not homogeneous. The career path—often spanning more than two decades—covers various career stages, each involving different professional and emotional demands (Huberman, 1989), and the roles—from classroom teacher to department head to school principal—impose varying emotional and social burdens. All of this supports the assumption that career stage and position may be substantive antecedents of profile membership (RQ2). However, the vast majority of existing literature on teachers’ EI is variable-centered and treats individual outcomes in isolation; a profile-based, heterogeneity-sensitive analysis of this population is still lacking.

### 2.4. Emotional–Social Intelligence, School Organizational Climate, and Conflict Management: Theoretical Expectations

We examine two of the organizational correlates of teachers’ emotional–social intelligence, as these are theoretically closely linked to emotional functioning and are of outstanding practical significance: the perception of the school organizational climate and the dominant conflict management style.

The school’s organizational climate—that is, the extent to which teachers perceive the institution as supportive, democratically led, attentive, collegially cohesive, and conducive to self-actualization—is not a directly objective organizational fact, but rather a perceived characteristic that is also shaped by the observer’s emotional–social dispositions (Hoy & Miskel, 2013). Educators with greater emotional competence are more sensitive to supportive aspects of leadership behavior, regulate frustration more effectively, and are more inclined to constructively reinterpret situations, which may be associated with a perception of a more supportive climate, particularly along the collegial and relational dimensions, which are most closely linked to interpersonal emotional skills. The empirical evidence—which is predominantly variable-centered—is consistent with this: the emotional intelligence of teachers and school leaders is positively associated with teachers’ climate perceptions and organizational trust (Allred et al., 2016; Chaudhary et al., 2024). According to a systematic review by Gómez-Leal et al. (2021) covering 35 studies, emotional intelligence is key to effective school leadership—the most frequently identified skills are self-awareness, self-regulation, and empathy—and that leaders’ trust-building relationships contribute significantly to teachers’ satisfaction and performance. From this, we hypothesize (H3a)—that teachers with a more adaptive, higher emotional–social profile will perceive the school’s organizational climate as more supportive.

In the field of conflict management, the Thomas–Kilmann model—following the logic of “dual concern”—distinguishes five styles along the two axes of self-assertion and cooperation: competition, problem-solving (integration), compromise, avoidance, and accommodation (K. W. Thomas & Kilmann, 1974; see Rahim, 2002). The conflict literature consistently associates cooperative, integrative styles with better relationship and performance outcomes, while avoidance and purely competitive strategies are typically associated with less favorable outcomes (De Dreu & Weingart, 2003). Theoretically, higher levels of emotional–social intelligence are associated with constructive styles, as these require emotional self-regulation, understanding the other person’s perspective, and flexible handling of the situation. In contrast, the avoidant style is typically associated with avoiding emotional confrontation, while the adaptiveness of accommodation is more context-dependent: it can be destructive if it involves self-sacrifice, but it can also be constructive if it serves to preserve the relationship or involves flexibility appropriate to the situation. Empirical evidence supports this: Valente and Lourenço (2020) used a structural equation model to demonstrate, based on a sample of 382 Portuguese teachers, that teachers with higher emotional intelligence employ more integrative and compromise-seeking strategies and thus handle classroom conflicts more constructively; this pattern is also supported by further studies in school and leadership contexts (Skordoulis et al., 2020; Shkëmbi & Treska, 2024; Millondaga et al., 2024; Völker et al., 2025). This leads to our hypothesis (H3b) that individuals with more adaptive profiles are more likely to exhibit a problem-solving dominant conflict style and less likely to exhibit an avoidance-dominant conflict style.

However, we note two theoretical considerations in advance. First, since both climate and conflict perceptions are self-reported and distal outcomes determined by many factors, the differences between profiles are expected to be of small-to-moderate strength; the contribution of the person-centered approach lies not in large effect sizes, but in identifying which configurations are associated with different organizational experiences. On the other hand—and this relates directly to the burden of proof outlined in Section 2.2—it is precisely these outcomes that determine the strength of the person-centered claim: if the profiles differ along the dimensions of climate and, in particular, conflict in ways that cannot be predicted by mere rank order, this supports the configuration-based, pattern-oriented interpretation; if, on the other hand, the differences follow a single level gradient, we must interpret this honestly in the Discussion and base our contribution on the descriptive power of the profiles, as well as on the practical significance of the climate and conflict gradients.

The research questions and hypotheses of this study are summarized below.

**RQ1.** *What distinct emotional–social intelligence profiles can be identified among educators?* **H1.** *We expect to find several distinct profiles that are primarily organized at the global level (elevation)—in line with the strong general factor of emotional–social intelligence—but that also exhibit certain configurational differences due to the distinguishability of the broad dimensions.*

**RQ2.** *Is profile membership correlated with position, career stage, and gender?* **H2.** *We are examining this question in an exploratory manner, as the various roles and career stages within the teaching profession may entail different emotional–social requirements, but the existing literature does not provide a sufficiently strong basis for formulating a directed hypothesis.*

**RQ3a.** *Do the profiles differ in terms of the perceived school organizational climate?* **H3a.** *Teachers belonging to the more adaptive (higher) profile perceive the school organizational climate as more supportive.*

**RQ3b.** *Do the profiles differ in terms of dominant conflict management style?* **H3b.** *The more adaptive profiles are more likely to exhibit a problem-solving-dominant conflict management style and less likely to exhibit an avoidance style.*

## 3. Methods

### 3.1. Sample and Procedure

A total of 587 teachers from 26 Hungarian public education institutions participated in the study; the sampling was institution-based and a convenience sample. The sample included staff members working in teaching positions, including those who also held leadership roles: 442 teachers, 68 leaders, and 43 school principals or vice principals (in one case, the position could not be validly determined). The average age of the participants was 44.6 years (*SD* = 9.2), and the average number of years in the profession was 21.1 (*SD* = 10.7), indicating a broad career trajectory spanning multiple career stages. Reflecting the gender composition of the teaching profession, the sample consisted predominantly of women (460 women, 85 men; in 42 cases, gender was not recorded). The number of participants per institution ranged from 7 to 46 (median 17), so the data form a multilevel structure embedded within institutions, which we treated separately during the analysis (see Section 3.3). The total number of participants far exceeds the recommended sample size for latent profile analysis and allows for the reliable identification of even smaller profiles (5–10%) (Nylund-Gibson & Choi, 2018; Spurk et al., 2020).

Data collection was conducted with the prior permission of the heads of the participating institutions, in group settings, using a paper-based questionnaire package. Prior to completing the questionnaire, participants were informed about the purpose of the research, the data collection process, the conditions of voluntary participation, the assurance of anonymity, and the main aspects of data management. Participation was voluntary, and the completion of the questionnaires can be interpreted as informed consent. The questionnaires did not contain any personally identifiable information, and the responses were analyzed exclusively in aggregated form. The study was conducted in accordance with the ethical principles of the Declaration of Helsinki.

### 3.2. Measurement Instruments

#### 3.2.1. Bar-On Emotional Quotient Inventory (EQ-i)

Emotional and social intelligence was measured using the validated Hungarian version of Bar-On’s (2006) EQ-i questionnaire. The questionnaire consists of 121 items, which respondents rate on a five-point Likert scale, and is organized into 15 subscales. The subscales form five main composites: (1) intrapersonal skills (self-confidence, emotional self-awareness, self-esteem, independence, self-actualization); (2) interpersonal skills (empathy, social responsibility, interpersonal relationships); (3) stress management (stress tolerance, impulse control); (4) adaptation (reality testing, flexibility, problem-solving); and (5) general mood (optimism, happiness). Subscale scores were calculated as the sum of the corresponding items, and composite scores were calculated as the average of the constituent subscales. We report internal consistency using both Cronbach’s alpha and McDonald’s omega, as the latter introduces less bias when item loadings are not identical and provides a more appropriate estimate for a congeneric measurement model (McDonald, 1999; McNeish, 2018). In the present sample, the internal consistency of the composites was adequate: intrapersonal α = 0.92 (ω = 0.92), interpersonal α = 0.89 (ω = 0.90), stress management α = 0.76 (ω = 0.77), adaptation α = 0.84 (ω = 0.85), and general mood α = 0.85 (ω = 0.87). Scoring followed the procedure of the validated Hungarian adaptation. Cronbach’s alpha and McDonald’s omega coefficients for all 15 subscales, computed under a uniform item-level method, are reported in Table S5 of the Supplementary Materials. The five composites served as indicators for latent profile analysis (see Table 1).

#### 3.2.2. Halász Organizational Climate Questionnaire

We measured perceptions of the school’s organizational climate using Halász’s (1980) organizational climate questionnaire, which contains 75 items on a five-point scale and is organized into eight theoretical dimensions: emphasis on performance, directness and democracy of leadership, personal leadership influence, attentiveness, the teaching staff as a work community, the teaching staff as a social group, close relationships, and freedom of self-actualization. The instrument thus captures the school’s organizational climate holistically: in addition to dimensions related to leadership, it also encompasses horizontal relationships within the teaching staff and the experience of individual self-actualization. The instrument was developed within early Hungarian research on school organizations as one of the first systematic attempts to measure school climate in Hungarian public education, and it has been used in Hungarian educational research since; because it is less known internationally, we summarize its structure and its psychometric properties in the present sample here. The eight dimensions were strongly correlated in the present sample (the average correlation among the dimensions was *r* = 0.62), and exploratory factor analysis indicated a clear one-factor structure: the first eigenvalue was 5.34, while all subsequent eigenvalues remained below 1 (the second eigenvalue was 0.88), and the first factor explained approximately 67% of the variance. Accordingly—and since the emotional intelligence composites were uniformly related to the eight dimensions, with no substantial differentiation among them—we operationalized the school organizational climate as a single, global “supportive climate” composite, calculated as the average of the eight dimensions. The reliability of this composite was excellent (α = 0.93). The climate was treated as a continuous outcome variable in the analysis.

#### 3.2.3. Thomas–Kilmann Conflict Management Questionnaire (TKI)

Conflict resolution style was measured using the Thomas–Kilmann Questionnaire (TKI), which consists of 30 forced-choice statement pairs and distinguishes five modes along the axes of self-assertion and cooperation: competing, collaborating/problem-solving, compromising, avoiding, and accommodating (K. W. Thomas & Kilmann, 1974). Due to the design of the instrument, it is ipsative: the respondent is forced to choose in each statement pair, so the sum of the five mode scores is nearly constant (≈30 in the present sample), and there is an artificial, negative correlation among the scores. Ipsative data cannot be interpreted as a standard set of independent, continuous variables, since the constrained covariance structure distorts intercorrelations, the factor structure, and criterion validity (Hicks, 1970; Cornwell & Dunlap, 1994; Brown & Maydeu-Olivares, 2013). We therefore analyzed conflict handling in two complementary ways. The primary analysis treated the five mode scores as a composition (Aitchison, 1986): cases whose row sum deviated from 30 were excluded (43 cases; remaining n = 542), zeros were handled by multiplicative replacement (δ = 0.5; Martín-Fernández et al., 2003), and the closed composition was transformed to centred log-ratios (CLR), on which profile differences were tested with one-way ANOVAs and Tukey contrasts, orthogonalized linear and quadratic trend tests, and a MANOVA on isometric log-ratio coordinates (see Compositional Analysis of the Complete Conflict-Mode Profiles Section; Tables S14–S18). As a secondary, descriptive analysis, we retained the dominant style—the mode with the highest score—as a categorical outcome; tied maxima (118 cases) were resolved by a fixed column order, and the consequences of this arbitrary rule are quantified in a dedicated robustness analysis (Section 4.7; Tables S6–S13).

#### 3.2.4. Professional and Demographic Characteristics

We included position, career stage, and gender as antecedents of profile membership. We coded position as a dichotomous variable based on leadership role (teaching staff vs. leadership, where the latter includes leaders and school principals/vice principals). We represented career stage using a continuous variable based on the number of years in the profession. Although we recorded age, we did not include it in the analysis because it correlated almost perfectly with the number of years in the profession (*r* = 0.91); thus, including both variables would have caused severe multicollinearity.

### 3.3. Analysis Strategy

The analysis was performed in Python 3.12 using the stepmix package (version 3.0.0), which provides a unified framework supporting latent profile analysis and one-, two-, and three-step unbiased procedures for linking profiles to external variables (S. Morin et al., 2025); inferential statistics were computed with SciPy and statsmodels (0.14.6). To ensure reproducibility and reduce the risk of local maxima, we estimated each model with a fixed initial value and multiple random restarts (Hipp & Bauer, 2006).

During the preprocessing stage, we examined descriptive statistics, distributions, and internal consistency; we mapped the patterns of missing data; and then we standardized the five EQ composites (*z*-scores). We accounted for missing data using the Full Information Maximum Likelihood (FIML) approach under the assumption of Missing at Random (MAR), so the analysis was based on the entire sample of 587 participants (Enders, 2010).

The main analysis was latent profile analysis, which we estimated for the five standardized composites, ranging from one to six classes; in the measurement model, we allowed the variance of the indicators to vary freely by class, which is a more flexible specification but requires more parameters (Masyn, 2013; Pastor et al., 2007). We selected the number of classes—in accordance with regional recommendations—by combining several criteria, since no single indicator is infallible: the minimum of the Bayesian (BIC) and sample-size-corrected Bayesian information criteria (aBIC), the bootstrap likelihood ratio test (BLRT), classification entropy (which we treated not as a fit measure but as a classification-discrimination measure; a value of ≥0.80 is desirable), the size of the smallest class (with a ≥5% threshold), and theoretical interpretability (Nylund et al., 2007; Tein et al., 2013; Nylund-Gibson & Choi, 2018; Spurk et al., 2020). We described the profiles of the selected solution using the class-wise averages of the indicators and named them based on their content.

We estimated the relationship between profiles and external variables using a three-step procedure. This approach is justified because, in a one-step model, the outputs feed back into the measurement model and may skew the meaning of the categories, while the traditional “classify-then-analyze” (modal) procedure—by ignoring classification uncertainty—systematically underestimates the relationship between profiles and external variables (Bolck et al., 2004; Vermunt, 2010). The unbiased, weighted BCH procedure (named after the work of Bolck et al., 2004) eliminates precisely this bias and has proven robust in simulations for both continuous and categorical outcomes (Asparouhov & Muthén, 2014; Bakk et al., 2013; Bakk & Vermunt, 2016). Accordingly, we examined the antecedents of profile membership (RQ2) using a three-step covariate model in which job classification, career stage, and gender jointly predicted profile membership (multinomial logistic regression with odds ratios). We also analyzed the distal outcomes (RQ3) using a three-step BCH correction procedure: the continuous school organizational climate using class-level estimated means and the Wald test for equality, and the categorical dominant conflict style using class-level probability estimates (categorical BCH). We thus used the modal “classify-then-analyze” approach only as a robustness check.

Finally, we verified the multilevel structure and the robustness of the solution in several ways. We calculated the intra-class correlation coefficient (ICC) for the school organizational climate at the institutional level, and—since ignoring clustered data can skew standard errors—we also validated the climate results using a mixed-effects model that included an institutional random effect (Hox et al., 2017; McNeish & Stapleton, 2016). The full multilevel mixed model (Vermunt, 2003) is theoretically more elegant but less stable given the relatively small number of clusters (26); therefore, we treated the institutional level as a random effect and conducted a sensitivity analysis rather than treating it as a separate latent level. We tested the stability of the profile structure by conducting a repeated analysis excluding the smallest institutions. In addition—to demonstrate the convergence of the person-centered and variable-centered perspectives—we also ran variable-centered regression models in which the five EQ composites directly predicted the school’s organizational climate and the dominant conflict style.

## 4. Results

The analyses were conducted in Python using the stepmix package (a continuous, FIML-like missing value handling measurement model; 3-step BCH and covariance procedures for the external variables); each model was estimated with fixed initial values and twenty restarts. The total sample consisted of n = 587 participants from 26 institutions.

### 4.1. Preliminary Analyses

Table 2 presents the descriptive statistics and inter-composite correlations for the five emotional–social intelligence composites. The composites are moderately to strongly correlated (r = 0.34–0.76; the strongest being between the intrapersonal and general mood composites, and the weakest between the interpersonal and stress management composites), which suggests the presence of a common emotional–social component while also indicating the empirical distinctiveness of the dimensions.

The institutional embeddedness of the school organizational climate is significant: based on the empty linear mixed model, 19.0% of the variance in the climate is attributable to differences between schools (ICC = 0.190); therefore, we confirmed the climate results not only through profile analysis but also using a mixed model that includes an institutional random effect (see Section 4.5).

### 4.2. Determining the Number of Profiles (RQ1)

We estimated latent profile solutions for the five standardized composites from one to six classes; the fit indices are summarized in Table 3. Neither the Bayesian information criterion (BIC) nor the sample-size-corrected Bayesian information criterion (aBIC) exhibited a local minimum: both decreased monotonically up to six classes, which is a common phenomenon in latent profile analysis. We therefore selected the solution based on a combination of several criteria. Significant improvements in the BIC occurred at the transitions from one to two, three, and four classes (ΔBIC = −672, −247, −101), after which the improvement leveled off markedly (four to five: −15.7; five to six: −5.5), indicating a clear inflection point at four classes. Furthermore, in the four-class solution, the smallest profile accounted for 9.4% of the sample, while this figure decreased to 6.5% for five classes and to 2.0% for six classes (the latter being non-interpretable); the classification entropy was adequate at four classes (0.80), and the profiles were well distinguished in terms of content. Taken together, these considerations supported the four-profile solution.

The bootstrap likelihood ratio test, computed by parametric bootstrap, rejected the K − 1 class solution at every step up to six classes (all *p* < .01; Table S1); like the information criteria, it therefore did not identify a stopping point by itself. The classification quality of the four-profile solution was adequate: the average posterior probabilities were 0.85, 0.86, 0.90, and 0.93 for P1–P4, respectively (Table S2).

### 4.3. Description of the Four Profiles

**Figure 1:**
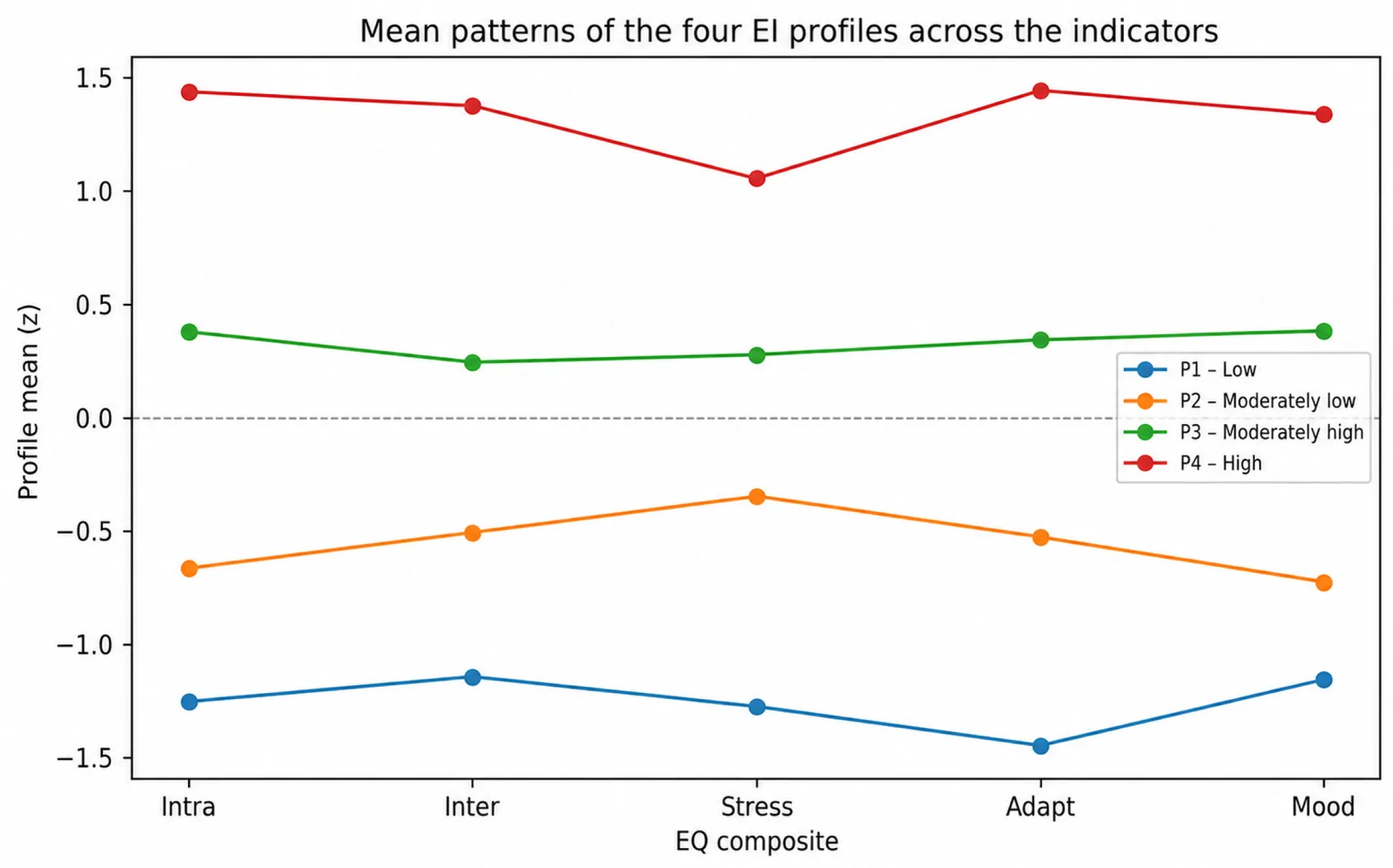
Average scores for the four EI profiles across the five indicators (z-scale).

Table 4 shows the dimensions of the four profiles and the average scores for each indicator (in both standardized and raw forms), while Figure 1 illustrates the average patterns of the profiles. The profiles differ primarily at the global level (elevation), ranging from a uniformly low profile (P1) to a uniformly high profile (P4), with a medium-low profile (P2) and a medium-high, balanced profile (P3)—which defines the sample and accounts for more than half of it—in between. In addition to the elevation pattern, moderate morphological variation can also be observed: the stress management composite is relatively lower (z = 1.06) in the highest profile (P4) compared to the other indicators, which range from 1.3 to 1.5 there; in other words, even the most developed profile is the least prominent in terms of stress management. The results partially support H1: several well-distinct profiles emerged, but these differ primarily in elevation; the structural variation is moderate and is mainly reflected in the relative lag in stress management in the highest profile.

A formal similarity assessment supported the elevational reading: Tucker’s congruence between the profiles’ standardized mean vectors was |φ| ≥ 0.965 for every pair (Table S3), the first principal component accounted for 64.6% of the variance of the five composites, and an exploratory bifactor model attributed 71% of the common variance of the 15 subscales to the general factor (ECV = 0.71, ω-hierarchical = 0.88; Table S4). The four-profile solution is therefore best read as a descriptive segmentation of a strongly general-factor-dominated construct rather than as evidence of qualitatively distinct configurations.

### 4.4. Antecedents of Profile Membership (RQ2)

We examined the predictors of profile membership using multinomial logistic regression (reference: the normative, moderately high P3 profile; predictors: leadership position, years in the field, gender; n = 546). The model as a whole did not reach significance (LR test *p* = .077; McFadden pseudo-R^2^ = 0.012), and no single predictor showed an effect at the *p* < .05 level (Table 5). Profile membership was thus essentially independent of the demographic and professional characteristics examined. The only consistent (albeit nonsignificant) trend was that leaders were less likely to belong to the lower profiles compared to the balanced P3 profile (P1 vs. P3: OR = 0.54; P2 vs. P3: OR = 0.63), suggesting that a leadership role is more closely associated with a more balanced emotional–social profile. Exploratory analyses of RQ2 indicated that profile membership was essentially independent of leadership position, career stage, and gender; the leadership role was associated with more adaptive profiles only as a weak, statistically insignificant trend.

### 4.5. School Organizational Climate (RQ3a)

**Figure 2:**
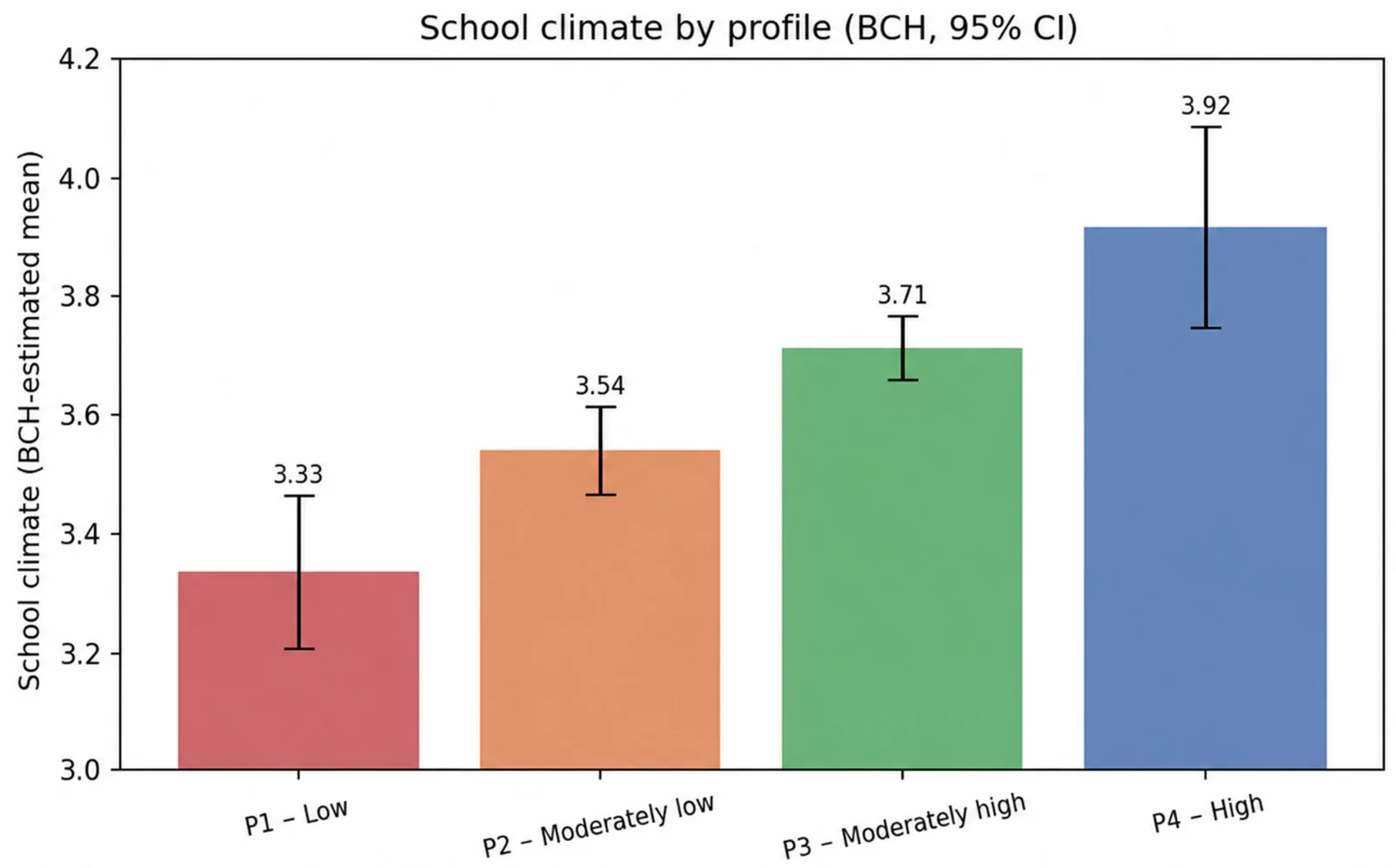
BCH-estimated profile averages for the school organizational climate, with a 95% confidence interval.

Table 6 and Figure 2 show the BCH-estimated averages of the school organizational climate by profile. Perceptions of the climate increase monotonically from lower to higher profiles (3.34 → 3.54 → 3.71 → 3.92 on a five-point scale). The difference between the profiles is statistically significant (one-way ANOVA: F(3, 583) = 15.34, *p* < .001, η^2^ = 0.073, small–medium effect). The mixed model examining institutional embeddedness (Climate ~ Profile + (1|School)) confirmed this same gradient (unstandardized coefficients, five-point scale): the medium high (b = 0.30, *p* < .001) and high (b = 0.54, *p* < .001) profiles both perceived a significantly more favorable climate than the low profile, while the difference between the medium and low profiles was marginal (b = 0.13, *p* = .059). The implied means from the mixed model (3.43/3.56/3.73/3.97) closely matched the BCH estimates, indicating that the climate outcome is robust to school embeddedness. Taken together, this monotonic gradient—robust to institutional embeddedness—supported H3a.

### 4.6. Conflict Management Style (RQ3b)

**Figure 3:**
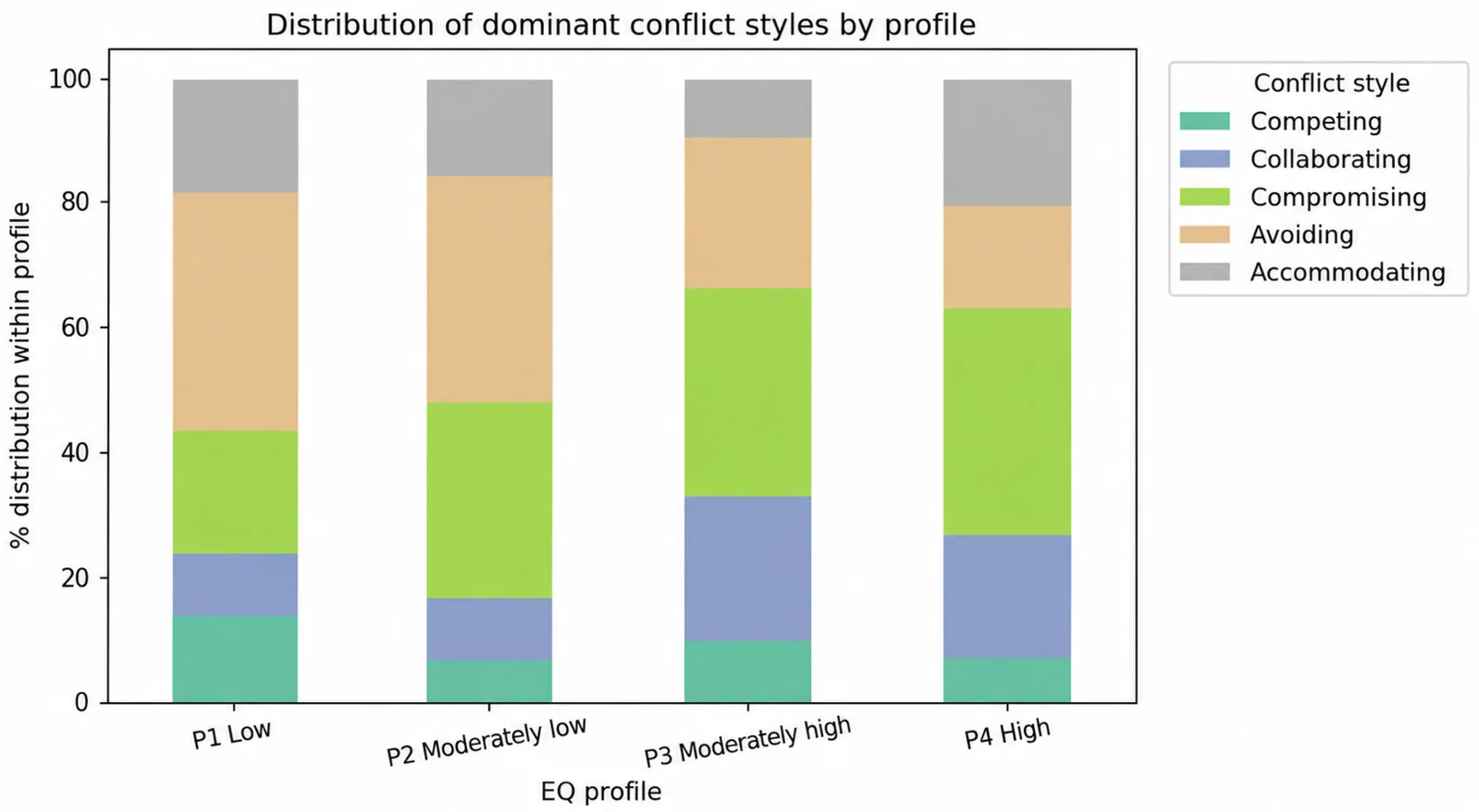
Distribution of dominant conflict management styles by profile.

The dominant conflict management style for each participant was determined by the mode with the highest Thomas–Kilmann score; in two cases, this was not clear, so this analysis was based on N = 585 individuals (the four-profile structure was reproduced unchanged in this subsample). The BCH-estimated probabilities of the dominant styles per profile are shown in the upper part of Table 7, while the observed contingency table is shown in the lower part; the distributions are illustrated in Figure 3. The difference between the profiles is significant (χ^2^(12) = 37.24, *p* < .001, Cramér-V = 0.146, small–medium effect; no expected cell frequency was below 5).

The proportion of teachers whose dominant style is avoiding decreases monotonically (BCH-estimated probabilities: 38.5% → 37.0% → 23.7% → 15.7%), while a dominant problem-solving style is more prevalent in the two higher profiles (P1/P2 ≈ 9–10% vs. P3 24.6%, P4 19.6%; Table 7). All percentages in this section refer to the proportion of persons assigned to a single dominant style and are therefore not numerically comparable with the compositional centroids reported in Table 8, which express the average relative weight of each mode within the complete five-mode profile (for problem-solving, 22.3% in P3 and 23.0% in P4). Because the dominant-style outcome discards information about relative intensities and, in the case of tied maxima, depends on an arbitrary resolution rule, we do not interpret finer differences at this level: the compositional analysis reported in Compositional Analysis of the Complete Conflict-Mode Profiles Section serves as the primary analysis of the conflict-mode pattern, and Section 4.7 quantifies the robustness of the dominant-style results.

#### Compositional Analysis of the Complete Conflict-Mode Profiles

The compositional analysis was conducted on the n = 542 cases with complete, constant-sum mode scores (P1 = 66, P2 = 138, P3 = 289, P4 = 49); profile membership was fixed from the primary solution. Table 8 shows the profile centroids in closed form. The relative weight of problem-solving rose from the lower profiles to P3 and then plateaued: the omnibus test was significant (F(3, 538) = 4.58, *p* = .004, η^2^ = 0.025; Holm-adjusted *p* = .007), the linear trend was significant (b = 0.065, *p* = .002) while the quadratic component was not (*p* = .55), and the P3–P4 contrast was null (Δ = 0.011, Tukey *p* = .997, d = 0.03). The relative weight of avoiding decreased with profile elevation (F(3, 538) = 9.49, Holm-adjusted *p* < .001; linear trend *p* < .001), although P1 and P2 did not differ. Accommodating carried greater relative weight in the two lower profiles than in P3 and P4 (F(3, 538) = 7.15, Holm-adjusted *p* < .001), with no P3–P4 difference (d = −0.06). Competing showed a genuinely curvilinear, U-shaped pattern (linear *p* < .001; quadratic *p* = .026), with its minimum at P2; compromising did not differ significantly across profiles after Holm adjustment.

The omnibus MANOVA on isometric log-ratio coordinates confirmed that the conflict-mode composition differs across profiles (Wilks’ Λ = 0.905, F(12, 1415.8) = 4.55, *p* < .001, partial η^2^ = 0.033). The closest profile centroids were P3 and P4 (Aitchison distance = 0.237), and the most distant were P2 and P4 (0.622), indicating that the conflict-mode space separates a lower (P1–P2) and an upper (P3–P4) block. Detailed tables are provided in the Supplementary Materials (Tables S14–S18).

### 4.7. Robustness Analyses

We verified the stability of the profile structure and the validity of the results in several ways. (a) Small institutions. There were no schools with fewer than 7 teachers in the sample; after excluding institutions with fewer than 10 teachers (2 schools, 15 teachers; remaining n = 572), the four-profile structure was reproduced unchanged (the z-means of the profiles are virtually identical, with the smallest profile at 11.7% and entropy at 0.80). (b) Variable-centered comparison. Multiple linear regression modeled on the school organizational climate (R^2^ = 0.141, *p* < .001) confirmed that emotional intelligence predicts climate perception (the strongest individual predictor is the interpersonal composite, std. β = 0.27); however, due to the strong correlation among the composites, multicollinearity was severe (VIF = 98–196), which limits the interpretability of the individual partial coefficients and, at the same time, supports the advantage of a person-centered approach that models the combined pattern of the composites. The direction of the multinomial logistic regression modeled on the dominant conflict style was consistent with the profile results (higher intrapersonal skills were associated with a lower probability of avoidance), while the nonlinear, saturating pattern of problem-solving identified in the compositional analysis was only partially reflected in the linear model. The two paradigms thus converge, reinforcing the validity of the results, while the person-centered approach adds a holistic interpretation of the combined patterns of the composites.

(c) Robustness of the dominant-style results. A full re-estimation bootstrap (B = 2000 replications, each comprising resampling, re-standardization, re-estimation of the four-profile model, and elevation-based relabeling), a leave-one-out jackknife over the members of P4, and a tie-breaking sensitivity analysis (original column order, exclusion of the 118 tied cases, and 1000 random resolutions) yielded a consistent picture (Tables S6–S13): the profile × dominant-style association remained significant in every variant; the higher problem-solving share of P4 relative to P1 was stable under resampling (95% CI [1.5; 29.1]), whereas the apparent accommodating surplus of P4 was not—the P4 − P3 difference included zero under the bootstrap (95% CI [−2.6; 20.9]), and the fixed column order proved to deflate the accommodating share in P1–P3 (accommodating reached the tied maximum in 55 cases and won none of them) while leaving P4 unaffected. The P3 − P4 difference in problem-solving was centered on zero under resampling (95% CI [−13.7; 12.7]), consistent with the compositional plateau.

### 4.8. Replication, Common-Method Variance, and Sensitivity Analysis

We addressed the three concerns arising from the cross-sectional, self-report design through targeted analyses. (a) Replication of the profile structure. By randomly splitting the sample into two halves twenty times, we estimated the four-profile solution separately for each half; the Tucker congruence of the average patterns of profiles paired according to elevation order was φ = 0.953 on average (median 0.96; range 0.90–0.98); every replication exceeded the 0.85 threshold (“adequate similarity”), and 70% of them exceeded 0.95, which indicates a practically identical structure (Lorenzo-Seva & ten Berge, 2006). The four-profile structure is thus reproducible even on independent subsamples. In addition, four-profile solutions estimated separately by gender reproduced the full-sample structure: in the female subsample (n = 460) the congruence was near-exact (Tucker’s φ = 0.983–0.998), while the male subsample (n = 85) was descriptively congruent (φ ≥ 0.96), although that solution lies near the feasibility limit of the model (Supplement Section S5, Table S19). (b) Common-method variance (CMV). In Harman’s one-factor test (Podsakoff et al., 2003), the first factor fitted to the 196 self-report items explained only 16.5% of the variance (well below the 50% threshold). In a confirmatory model including a common method factor (CLF), the latent correlation between emotional intelligence and school organizational climate changed only slightly (0.26 to 0.23) when controlling for the common method factor and remained significant (*p* < .001). According to these two consistent procedures, common-method variance is not a dominant source of bias; moreover, the conflict-outcome scale uses a forced-choice (ipsative) format, which differs from Bar-On’s Likert items and thus structurally limits the role of common method in this relationship. (c) Sensitivity to unmeasured confounders. The E-value (VanderWeele & Ding, 2017) calculated for the climate difference between the highest and lowest profiles (Cohen’s d = 0.88) is 3.89 (with a lower bound of 2.58 for the 95% confidence interval): an unmeasured confounder would need to have a relationship with both profile membership and climate that is approximately 3.9 times (or 2.6 times, according to a conservative estimate) as strong to fully explain the association—this exceeds the strength of typical demographic confounders.

Overall, these checks indicate that the main findings cannot be attributed to unstable profile formation, a dominant common-method bias, or any easily hypothesized unmeasured confounding effect. At the same time, due to the cross-sectional design, the results still cannot be interpreted as causal evidence; rather, they support robust, methodologically controlled associations between teachers’ emotional–social intelligence profiles and their perceptions of organizational functioning.

## 5. Discussion

The aim of the study was to map teachers’ emotional and social intelligence—following the dominance of the variable-centered tradition—using a person-centered approach, and to explore how the resulting profile structure relates to the antecedents of profile membership, as well as to perceptions of the school’s organizational climate and conflict management. The study met the burden of proof outlined in Section 2.2: not only are the profiles reliably distinguishable and reproducible across independent subsamples, but they also exhibit a nonlinear, saturating relationship with conflict management that a purely linear elevation logic would not predict. The identification of profiles and the exploration of their concurrent correlates is a matter of structural-descriptive and concurrent validity, for which a cross-sectional design is appropriate; however, we do not claim a causal direction. The results partially supported H1: several well-distinct but predominantly elevation-based profiles emerged. The findings supported H3a; H3b received partial support—clearly in the form of a lower rate of avoidance, and in the rising-then-saturating pattern of problem-solving. According to the exploratory responses to RQ2, profile membership was essentially independent of leadership position, career stage, and gender. We will elaborate on this pattern below.

### 5.1. Interpretation of the Four Profiles

Based on Bar-On’s five composites, four distinct profiles emerged that were well replicated across independent subsamples; these differ primarily at the global level: a uniformly low profile (P1, 12.4%), a medium-low profile (P2, 25.4%), a balanced, medium-high profile that defines the sample (P3, 52.8%), and a uniformly high profile (P4, 9.4%). Thus, more than half of the teachers belong to a single, balanced middle group, which is flanked by a smaller group with fewer emotional and social resources and a similarly sized, high-performing group. This structure is informative in and of itself: it suggests that the teaching community is not homogeneous from an emotional–social perspective, nor does it break down into many sharply distinct types, but rather is organized around a dominant, normative core. Since the profiles were derived from self-reported composites, and the Bar-On model measures self-perceptions of emotional–social competence rather than maximum performance (see Section 2.1), we interpret these profiles as self-perceived resource patterns.

### 5.2. Elevation and Configuration: The Benefits of a Person-Centered Approach

The predominantly elevation-based organization of the profiles raises, at first glance, the question we ourselves anticipated in Section 2.2: if the groups are essentially intersections of a single continuum of “more or less emotional–social capacity,” what does the profile approach add? The answer consists of two complementary parts. First, the dominance of elevation is not a shortcoming but a theoretically expected and meaningful result: it is consistent with the fact that a strong general factor underlies emotional abilities (Simonet et al., 2021) and that the Bar-On composites are strongly correlated in the present sample as well (r = 0.34–0.76). The profile structure thus reliably captures the heterogeneity in the levels of emotional–social resources and provides an informative picture of the internal structure of the teaching community: it is organized around a dominant, balanced core, framed by two extreme groups with opposite polarities.

However, the substantive advantage of the person-centered approach is evident in the outcomes of conflict management, and it is worth specifying exactly what that is. A clear elevation gradient would predict a monotonic relationship for every conflict style; the data only partially support this. The proportion of the avoiding style does indeed decrease monotonically with profile level (≈38% → 37% → 24% → 16%)—this is the clearest and most robust support for H3b. The compositional analysis specifies the shape of this association: the relative weight of problem-solving rises from the lower profiles to P3 and then plateaus—P3 and P4 are statistically indistinguishable (d = 0.03) despite the large elevation difference between them—while competing follows a genuinely curvilinear, U-shaped pattern with its minimum at P2. This is the study’s central substantive contribution: the relationship between emotional–social intelligence and constructive conflict handling is not a simple “more ESI = more constructive style” linear relationship but a saturating one—beyond the modal profile, additional emotional–social resources are not accompanied by a further increase in the relative weight of problem-solving—a correction to the predominantly linear framing of the EI–conflict literature (Valente & Lourenço, 2020; Völker et al., 2025).

At the dominant-style level, the highest profile also showed a seemingly elevated share of accommodating dominance; this observation did not withstand robustness scrutiny, and we do not interpret it substantively. Three independent lines of evidence converged (Section 4.7): the fixed column order used to resolve tied dominant styles systematically deflated the accommodating share in P1–P3 while leaving P4 unaffected; the P4 − P3 difference was not stable under a full re-estimation bootstrap; and at the level of relative intensities the contrast disappeared entirely (18.1% vs. 18.3% of the compositional centroids). What the highest profile does show—the absence of a single prevailing dominant style—describes the distribution of dominance frequencies across individuals and licenses no inference about intra-individual repertoires. The robust nonlinearity is the one described above: monotonically decreasing avoidance and a rising-then-saturating pattern of problem-solving. Finally, the strong correlation among the composites (and the VIF values of 98–196 observed in the variable-centered model) demonstrates precisely why it is difficult to isolate individual facet effects in a linear model and why a person-centered approach that models the combined pattern of the composites is informative.

### 5.3. Antecedents, Effect Sizes, and Interpretive Framework

Profile membership was essentially independent of the demographic and professional characteristics examined: the multinomial model as a whole did not reach significance (*p* = .077; McFadden pseudo-R^2^ = 0.012), and no single predictor showed an effect at the *p* < .05 level; the only consistent (though nonsignificant) trend was that leaders tended to gravitate toward balanced, higher-scoring profiles. This null result is not a shortcoming but a meaningful finding: it indicates that the configuration of emotional–social intelligence does not stratify along demographic or role-based fault lines within the profession—thus, it cannot be automatically assumed that more experienced or senior educators are emotionally “more developed”—and that the profiles are not merely proxies for position or career stage.

The relationships between the profiles and organizational outcomes are of modest to moderate strength (climate: η^2^ = 0.073; conflict: Cramér-V = 0.146), which is the order of magnitude expected for self-reported, multifactorial distal outcomes. The climate result, however, is remarkably robust: the gradient is monotonic, survives the mixed model accounting for institutional embeddedness, and, according to the E-value (3.89), could only be fully explained by an unusually strong, unmeasured confounder. However, since we used a mixed-model, self-report EI measure, and climate is a perceived construct here, part of the gradient may also stem from dispositional positivity (see Section 6); the conflict-outcome measure, with its distinct, ipsative format, is structurally more robust in this regard.

The validity of the results is reinforced by the convergence of the two paradigms: variable-centered models also showed that higher, more adaptive emotional–social intelligence is associated with a more supportive climate perception and less avoidance. Facet-level details (such as the relative weights of individual composites) should be interpreted with caution due to severe multicollinearity; however, the overall trend is consistent across the two approaches.

### 5.4. Practical Implications

Approximately one-third of teachers (combining the P1 and P2 profiles) are characterized by self-reported emotional–social resources below the average, and this group perceives a less supportive school climate while also tending toward avoidant conflict resolution. Since emotional–social competencies can be developed, and targeted interventions are effective even among the teaching population (González-Ros et al., 2026), these profiles may represent an identifiable target group for teacher professional development—primarily in the areas of reducing avoidance, strengthening constructive conflict engagement, and stress management (the most distinct dimension). Given the cross-sectional, self-report design, we frame this as a promising direction for intervention research rather than as an evidence-based recommendation.

The variation among the profiles also highlights the need to refine the goal of professional development. The saturating pattern of problem-solving suggests that the aim of development is not to teach a single “correct” conflict style, but to reduce avoidance and support constructive engagement. The monotonic gradient of the climate and the (weak) tendency of leaders toward a more balanced profile suggest that both teachers’ and leaders’ emotional–social competencies can play a role in shaping a supportive school organizational climate; thus, leadership development also has an emotional–social dimension. In light of the modest effect sizes, all of this should be treated as a realistic but not exclusive factor in professional development.

From a theoretical perspective, this study extends the logic of person-centered, profile-based intelligence research to the emotional–social domain within a previously neglected population and organizational context, thereby enriching the existing, predominantly non-educational person-centered EI literature (Gabriel et al., 2015; Toyama & Mauno, 2016; Pirsoul et al., 2023). Although the identified profiles differed primarily in overall elevation, the results also contribute to our understanding of individual trajectories of emotional–social development: they suggest that adaptive functioning is associated with emotional resources not in a uniform, linear fashion, but through a saturating, non-linear pattern. Its most significant substantive contribution is the demonstration that the association between emotional–social intelligence and constructive conflict management is graded and saturating rather than uniformly linear---a pattern that a variable-centered, linear approach can capture only to a limited extent.

## 6. Limitations of the Study

The results should be interpreted in light of the following limitations; these qualify but do not invalidate the conclusions presented above.

First, the study is cross-sectional, self-report-based, and single-source; therefore, the relationships between profiles and organizational outcomes cannot be interpreted as evidence of causation. The replication, common-method, and sensitivity analyses presented in Section 4.8 mitigate but do not eliminate this limitation: the observed associations should be interpreted as robust correlations, not causal effects.

Second, and closely related to this, the mixed-model EI measurement—due to its overlap with personality and dispositional positivity—does not rule out the possibility that part of the climate gradient is a perceptual-dispositional halo effect (see Section 5.3). We did not measure personality traits (particularly neuroticism and extraversion), so we could not directly control for this confounding; common-method analyses and the E-value provide only partial protection, as they target common-method effects rather than trait confounding.

We regard the absence of personality controls—particularly neuroticism and extraversion—as the most important measurement-related limitation of the study; future studies should include them as covariates or rival predictors.

Third, the conflict-handling outcome required particular care. The dominant-style operationalization discards information, and our sensitivity analyses showed that its fixed tie-breaking rule systematically biased the accommodating shares; we therefore treated the compositional analysis as primary and interpreted the dominant-style results only where they proved robust to the operationalization (Tables S6–S13). The small size of the highest profile (P4, n = 55) nevertheless limits the precision of profile-specific estimates (bootstrap intervals of ten percentage points or more; Table S8), which calls for replication on independent samples.

Fourth, the latent profile solutions are sample-dependent, and the fit indices did not indicate a clear optimum: neither the BIC nor the aBIC reached a minimum, and entropy did not favor the four-class solution; the bootstrap likelihood ratio test likewise remained significant up to six classes (Table S1). The decision in favor of the four-profile model is thus a judgment based on the combined consideration of several criteria (elbow point, smallest profile size, interpretability), which we made in contrast to the five-profile alternative; this further underscores the need for independent replication. Relatedly, a formal multi-group test of profile-structure invariance across gender was not feasible given the size of the male subsample (n = 85, against 43 free parameters); the congruence-based check reported in the Supplementary Material (Section S5, Table S19) is descriptive only, and subgroup invariance should be established on larger samples.

Fifth, the profile estimation was one-level, while the institutional embedding of the climate was substantial (ICC = 0.19). Although we confirmed the climate results using a mixed model that included an institutional random effect, and excluding small institutions did not alter the profile structure, we did not test for potential institutional-level clustering of profile membership itself.

Sixth, due to the ipsative structure of the Thomas–Kilmann questionnaire, we operationalized conflict management using the dominant style; this loses information about the intensity and relative weight of the styles and—as explained in Section 5.2—is also structurally unsuitable for examining intra-individual stylistic flexibility.

Finally, the two subscales of the stress management composite showed relatively weak correlation (although the composite’s item-level α = 0.76 is adequate), which calls for caution in interpreting this dimension; the range of antecedents (position, career stage, gender) was narrow, and numerous potential predictors (personality, prior training, institutional characteristics) were omitted; furthermore, we treated the work climate as a single, empirically justified composite that obscured the finer distinctions among the eight dimensions. The composition of the sample (predominantly female Hungarian public school teachers) limits the generalizability of the findings to other cultural, institutional, and occupational contexts.

## 7. Conclusions

To the best of our knowledge, this is the first person-centered study to link teachers’ Bar-On emotional–social intelligence to two organizational correlates—perceptions of school organizational climate and conflict management—simultaneously, using a large, multilevel sample and unbiased three-step procedures. It yielded three mutually reinforcing findings. First, it identified a four-profile emotional–social structure that was reliably reproduced across independent random split-half subsamples, in each case organized around a dominant, balanced middle group. Second, teachers belonging to the higher, more adaptive profile perceive the school’s organizational climate as more supportive—along a monotonic gradient that is robust to both institutional embeddedness and unmeasured disturbances—and are clearly less avoidant in conflict management. Third, and this is the central novelty of this study, the relationship between emotional–social intelligence and constructive conflict handling proved to be nonlinear: the relative weight of problem-solving rises up to the modal, balanced profile and then plateaus—the highest profile shows no further gain—while competing follows a U-shaped pattern across profiles. This saturating, non-monotonic patterning is information that a variable-centered, linear approach can capture only to a limited extent.

The conflict-handling findings are best summarized as a graded and saturating association: the relative weight of problem-solving rises up to the modal profile and then levels off, avoidance declines with profile elevation, and the apparent accommodating surplus of the highest profile proved to be an artifact of the dominant-style coding and is therefore not interpreted. Furthermore, profile membership was essentially independent of the examined demographic and professional characteristics, and the associations with organizational outcomes—in line with the self-reported, distal outcomes—were of modest to moderate strength.

With this, the study extends person-centered, profile-based intelligence research to the emotional–social domain and provides a practical reference point: it identifies a subgroup of educators (those with lower profiles) who simultaneously perceive a less supportive climate and tend toward avoidant conflict handling and who are thus plausible candidates for targeted professional development built on developable emotional–social competencies. Testing this implication requires the longitudinal, multi-source, and intervention-based designs outlined in Section 6.

## Figures and Tables

**Table 1:** Characteristics of the Bar-On EQ-i composites and subscales, and the reliability of the school organizational climate composite (N = 587).

ω	α	SD	M	Item Number	Scale
0.92	0.92	3.28	26.21	36	**Intrapersonal Skills**
	0.60	–	–	6	Self-confidence
	0.77	–	–	7	Emotional self-awareness
	0.87	–	–	9	Self-esteem
	0.72	–	–	7	Independence
	0.73	–	–	7	Self-actualization
0.90	0.89	3.62	32.38	24	**Interpersonal Skills**
	0.72	–	–	5	Empathy
	0.77	–	–	9	Sense of social responsibility
	0.81	–	–	10	Interpersonal relationships
0.77	0.76	3.89	30.29	18	**Stress management**
	0.43	–	–	9	Stress tolerance
	0.85	–	–	9	Impulse control
0.85	0.84	3.47	31.66	26	**Adaptability**
	0.75	–	–	10	Reality testing
	0.71	–	–	8	Flexibility
	0.76	–	–	8	Problem-solving
0.87	0.85	4.09	31.47	17	**General mood**
	0.67	–	–	8	Optimism
	0.82	–	–	9	Happiness
0.93	0.93	0.59	3.65	75 (8 dim.)	**School organizational climate (global composite) ^a^**

Note. M = mean, SD = standard deviation; α = Cronbach’s alpha, ω = McDonald’s omega (calculated at the item level using a one-factor model). Composites are the mean of the subscales, and subscales are the sum of their respective items; M and SD refer to the raw scores of the composites. The relatively low internal consistency of the Stress Tolerance subscale (α = 0.43, ω = 0.53) is offset at the level of the 18-item Stress Management composite (α = 0.76, ω = 0.77). ^a^ The school organizational climate was measured using a single composite score calculated as the average of Halász’s eight dimensions (see Section 3.2.2); M and SD refer to the five-point scale. Due to the ipsative structure of the Thomas–Kilmann Conflict Management Questionnaire, traditional internal consistency cannot be interpreted and is therefore not reported here (see Section 3.2.3).

**Table 2:** Descriptive statistics and Pearson correlations for the five EI composites (N = 587).

5	4	3	2	1	SD	M	Composite
				—	3.28	26.21	1. Intrapersonal
			—	0.57	3.62	32.38	2. Interpersonal
		—	0.34	0.46	3.89	30.29	3. Stress Management
	—	0.63	0.55	0.63	3.47	31.66	4. Adaptation
—	0.56	0.40	0.61	0.76	4.09	31.47	5. General Mood

Note. All correlations are *p* < .01. Values refer to the raw scores of the composites.

**Table 3:** Fit indices for latent profile solutions with 1–6 classes (N = 587).

Smallest Class (%)	Entropy	AIC	aBIC	BIC	Free par.	logL	Classes (K)
100.0	1.000	7917.8	7929.8	7961.6	10	−3948.9	1
37.3	0.769	7197.7	7222.9	7289.6	21	−3577.9	2
18.4	0.777	6902.9	6941.3	7042.9	32	−3419.4	3
**9.4**	**0.802**	**6753.9**	**6805.5**	**6942.0**	**43**	**−3334.0**	**4**
6.5	0.812	6690.0	6754.9	6926.3	54	−3291.0	5
2.0	0.826	6636.4	6714.5	6920.8	65	−3253.2	6

Note. aBIC = sample-size-corrected (Sclove) BIC. The selected solution is shown in bold.

**Table 4:** Dimensions of the four EI profiles and the average scores for each indicator by profile (N = 587).

General Mood	Adaptability	Stress Management	Interpersonal	Intra (z/raw)	%	n	Profile
−1.15/26.76	−1.45/26.64	−1.27/25.34	−1.14/28.25	−1.25/22.11	12.4	73	P1—Low
−0.72/28.51	−0.53/29.83	−0.35/28.94	−0.51/30.55	−0.66/24.04	25.4	149	P2—Medium-low
0.38/33.03	0.34/32.85	0.28/31.38	0.25/33.27	0.38/27.45	52.8	310	P3—Medium-high
1.34/36.95	1.45/36.68	1.06/34.41	1.38/37.37	1.44/30.92	9.4	55	P4—High

Note. z = standardized mean; raw = mean calculated on the composite’s original scale.

**Table 5:** Antecedents of profile membership: multinomial logistic regression (reference: P3—Medium high; n = 546).

*p*	95% CI	OR	Predictor	Contrast
.114	[0.25, 1.16]	0.54	Leaders (vs. teacher)	P1—Low vs. P3
.070	[0.95, 1.00]	0.98	Years in the field	
.380	[0.38, 1.45]	0.74	Gender (female vs. male)	
.086	[0.37, 1.07]	0.63	Leaders (vs. teacher)	P2—Medium-low vs. P3
.610	[0.99, 1.02]	1.01	Years in the field	
.945	[0.58, 1.79]	1.02	Gender (female vs. male)	
.458	[0.34, 1.63]	0.74	Leaders (vs. teacher)	P4—High vs. P3
.097	[0.95, 1.00]	0.98	Years in the field	
.283	[0.66, 4.14]	1.65	Gender (female vs. male)	

Note. Pseudo-R^2^ (McFadden) = 0.012; LR test for the entire model, *p* = .077. The direction of the bias-adjusted 3-step covariance estimate was the same (the leadership coefficient was positive for P3 and negative for lower profiles).

**Table 6:** School organizational climate by profile: BCH estimate and mixed model (N = 587).

*p*	Mixed-Model: b (vs P1)	SE	BCH Mean	Profile
—	—(intercept = 3.433)	0.078	3.335	P1—Low
.059	+0.128	0.045	3.540	P2—Medium-low
<.001	+0.298	0.031	3.711	P3—Medium-high
<.001	+0.538	0.093	3.915	P4—High

Note. Omnibus: F(3, 583) = 15.34, *p* < .001, η^2^ = 0.073. ICC (school) = 0.190. The mixed model treats school embedding as a random intercept. Mixed-model coefficients (b) are unstandardized and expressed in the original units of the five-point climate scale, with P1 (Low) as the reference category; the model intercept (3.433) corresponds to the P1 estimate.

**Table 7:** Dominant conflict management style by profile (N = 585).

BCH-Estimated Probabilities (%).						
Accommodating		Avoiding	Compromising	Problem-Solving	Competing	Profile
18.8		38.5	17.5	9.7	15.4	P1—Low
16.1		37.0	32.1	9.0	5.9	P2—Medium-low
8.4		23.7	33.2	24.6	10.2	P3—Medium-high
21.0		15.7	36.6	19.6	7.0	P4—High
**Number of Units Observed (Percentage)**						
**Σ**	**Accommodating**	**Avoiding**	**Compromising**	**Problem-Solving**	**Competing**	**Profile**
71	13 (18.3)	27 (38.0)	14 (19.7)	7 (9.9)	10 (14.1)	P1—Low
147	23 (15.6)	53 (36.1)	46 (31.3)	15 (10.2)	10 (6.8)	P2—Medium-low
312	29 (9.3)	76 (24.4)	103 (33.0)	73 (23.4)	31 (9.9)	P3—Medium-high
55	11 (20.0)	9 (16.4)	20 (36.4)	11 (20.0)	4 (7.3)	P4—High

Note. χ^2^(12) = 37.24, *p* < .001, Cramér-V = 0.146. The BCH probabilities are estimates corrected for uncertainty in profile classification; the observed percent ranks are in good agreement with them. The numbers of participants per profile are derived from a four-profile solution re-estimated on a subsample of n = 585 (after excluding the two cases with predominantly unidentifiable conflict styles); therefore, the numbers per profile differ slightly from the results for the full sample (N = 587) (Table 4), due to the reclassification of a few borderline cases.

**Table 8:** Compositional centroids of the conflict-mode profiles by ESI profile (closed geometric means, %; n = 542).

Accommodating	Avoiding	Compromising	Problem-Solving	Competing	Profile
22.0	26.9	22.7	18.9	9.5	P1—Low
22.3	26.4	24.6	19.4	7.4	P2—Medium-low
18.3	23.8	25.5	22.3	10.2	P3—Medium-high
18.1	21.5	24.8	23.0	12.6	P4—High

**Note**. Closed geometric means of the five conflict modes after multiplicative zero replacement (δ = 0.5); rows sum to 100%. The values express the average relative weight of each mode within a respondent’s complete five-mode profile and are therefore not directly comparable with the dominant-style percentages in Table 7, which are proportions of persons.

## Data Availability

The data presented in this study are available on request from the corresponding author. The data are not publicly available because participants consented to the analysis of their responses within the framework of the original research only, and because the dataset contains school-level identifiers for 26 institutions that could permit the re-identification of participating schools.

## Supplementary Materials

The following supporting information can be downloaded at https://www.mdpi.com/article/10.3390/jintelligence14080184/s1: Section S1: Model selection and classification diagnostics (Tables S1 and S2); Section S2: Profile similarity and general-factor analyses (Tables S3–S5); Section S3: Robustness of the dominant-style analyses (Tables S6–S13); Section S4: Compositional analysis of the conflict-mode profiles (Tables S14–S18); Section S5: Subgroup congruence of the profile structure (Table S19); Section S6: Reproducibility.
