# Supplementary material for: Emotional–Social Intelligence Profiles and Adaptive Professional Functioning Among Teachers: A Person-Centered Analysis of School Climate Perception and Conflict Handling
Source: J Intell. 2026 Aug 10;14(8):184. doi: 10.3390/jintelligence14080184 (PMC13514884; doi:10.3390/jintelligence14080184)
Supplement: Supplementary file 1 [file jintelligence-14-00184-s001.zip › jintelligence-4414556-supplementary.pdf]

Supplementary Materials

Emotional–Social Intelligence Profiles and Adaptive Professional Functioning Among Teachers: A Person-Centered Analysis of School Climate Perception and Conflict Handling (Manuscript ID: jintelligence-4414556)

All analyses reported here were conducted in Python (StepMix 3.0.0 for latent profile estimation; SciPy/statsmodels for inferential statistics). Unless stated otherwise, latent profile models used five standardized Bar-On composites as indicators, a Gaussian measurement model with class-specific variances and full-information treatment of missing indicator values (no listwise deletion), 20 random initializations, a maximum of 1,000 EM iterations, and a fixed random seed (42). Profiles are labeled P1–P4 in ascending order of overall elevation.

S1. Model Selection and Classification Diagnostics

The bootstrap likelihood ratio test (BLRT) was computed by parametric bootstrap: for each comparison of K − 1 versus K classes (K = 2…6), 100 samples of N = 587 were generated from the fitted (K − 1)-class model, both models were re-estimated on each generated sample (5 random initializations for the null-distribution fits; 20 for the observed-data fits), and the observed likelihood ratio statistic was referred to the resulting null distribution. Generated samples were complete data matrices; the observed data contained a small number of missing indicator values handled by full-information maximum likelihood.

Table S1. Bootstrap likelihood ratio tests for the number of latent profiles (N = 587).

Comparison (K − 1 vs. K)	Observed LRT	95th percentile of null	Maximum of null	Bootstrap p
1 vs. 2	742.08	31.23	43.36	< .01
2 vs. 3	316.83	35.03	39.12	< .01
3 vs. 4	170.99	33.17	38.63	< .01
4 vs. 5	85.86	33.08	40.88	< .01
5 vs. 6	75.6	37.92	47.3	< .01

Note. B = 100 bootstrap samples per comparison. The observed statistics exceed the entire null distribution at every step; like the information criteria, the BLRT therefore does not identify a stopping point up to six classes, and the four-profile decision rests on the combination of criteria described in the main text (Section 3.3).

Table S2. Classification diagnostics of the four-profile solution (N = 587).

Profile	n	Average posterior probability (AvePP)
P1	73	0.853
P2	149	0.857
P3	310	0.903
P4	55	0.926

Note. Relative entropy = .802. All AvePP values exceed the conventional .80 threshold.

S2. Profile Similarity and General-Factor Analyses

Table S3. Tucker's congruence coefficients between the standardized mean-score vectors of the four profiles (N = 587).

	P1	P2	P3	P4
P1	1	0.965	-0.987	-0.988
P2	0.965	1	-0.985	-0.986
P3	-0.987	-0.985	1	0.982
P4	-0.988	-0.986	0.982	1

Note. Absolute congruence between all profile pairs is ≥ .965, indicating essentially collinear (elevation-differentiated) mean patterns. Consistently, the first principal component of the five composites accounts for 64.6% of their total variance.

Table S4. Exploratory bifactor model of the 15 EQ-i subscales (general factor + five orthogonal specific factors; complete cases, n = 455).

Subscale	General-factor loading	Largest specific loading	Uniqueness
Self-confidence	0.548	0.47	0.478
Emotional self-Awareness	0.778	-0.014	0.395
Self-esteem	0.815	0.144	0.315
Independence	0.527	0.668	0.277
Self-actualization	0.813	0.014	0.338
Empathy	0.456	0.819	0.122
Sense of social responsibility	0.49	0.514	0.495
Interpersonal relationship	0.763	0.291	0.334
Stress tolerance	0.629	0.08	0.598
Impulse control	0.344	0.08	0.875
Reality testing	0.61	0.76	0.05
Flexibility	0.54	0.066	0.704
Problem-solving	0.547	0.326	0.594
Optimism	0.733	0.254	0.399
Happiness	0.828	0.254	0.25

Note. Two-indicator specific factors (stress management, general mood) were identified with equality-constrained loadings; uniquenesses were bounded at ≥ .05. Model fit: $\chi^2(77)$ = 795.6, CFI = .821, RMSEA = .143. Explained common variance of the general factor (ECV) = .711; ω-hierarchical = .88. Given the modest global fit, the model is reported as an exploratory quantification of general-factor dominance rather than as a preferred measurement model.

Table S5. Internal consistency of the 15 EQ-i subscales and the stress management composite.

Scale	Items	Cronbach's α	McDonald's ω
Self-confidence	6	0.604	0.629
Emotional self-awareness	7	0.769	0.773
Self-esteem	9	0.873	0.874
Independence	7	0.717	0.726
Self-actualization	7	0.729	0.733
Empathy	5	0.725	0.733
Sense of social responsibility	9	0.768	0.781
Interpersonal relationship	10	0.814	0.826
Reality testing	10	0.75	0.758
Flexibility	8	0.709	0.718
Problem-solving	8	0.762	0.775
Stress tolerance	9	0.432	0.529
Impulse control	9	0.85	0.859
Optimism	8	0.668	0.759
Happiness	9	0.815	0.819
Stress management composite (stress tolerance + impulse control)	18	0.765	0.774

Note. The correlation between the stress tolerance and impulse control subscale scores is r = .382. Coefficients were computed on complete cases per scale; ω from single-factor maximum-likelihood models. Subscale labels follow Table 1 of the main text.

S3. Robustness of the Dominant-Style Analyses

The dominant conflict style (TK_dom) was defined as the mode with the highest TKI score; ties (n = 118 of 585; 20.2%) were originally resolved by a fixed column order (competing > problem-solving >

compromising > avoiding > accommodating). Three procedures assessed the robustness of the profile × dominant-style results: (i) a full re-estimation bootstrap (B = 2,000; each replication comprised resampling with replacement, re-standardization, re-estimation of the four-profile LPA on the N = 585 subsample, elevation-based relabeling, and recomputation of the profile × style table); (ii) a leave-one-out jackknife over the 55 members of P4 with fixed profile membership; and (iii) a tie-breaking sensitivity analysis comparing the original coding, exclusion of tied cases, and 1,000 random resolutions drawing uniformly among the tied modes.

Table S6. Composition of the 118 tied cases: participation and wins by conflict style under the original column-order rule.

Style	Reached the within-person maximum (n)	Won under column order (n)
Competing	16	16
Problem-solving	45	39
Compromising	64	37
Avoiding	79	26
Accommodating	55	0

Note. The 118 ties comprise 259 style participations (99 two-way, 16 three-way, 2 four-way, 1 five-way). Accommodating, last in the column order, participated in 55 ties and won none; the fixed order therefore systematically deflates its dominant-style share.

Table S7. Tied cases by profile: winning style under the original rule (upper panel) and styles involved in the ties (lower panel).

Profile	Competing	Problem-solving	Compromising	Avoiding	Accommodating
P1 — won	3	3	3	3	0
P2 — won	5	5	15	10	0
P3 — won	6	30	15	13	0
P4 — won	2	1	4	0	0
P1 — involved	3	3	5	9	5
P2 — involved	5	6	18	29	19
P3 — involved	6	33	36	36	31
P4 — involved	2	3	5	5	0

Note. Tied cases per profile: P1 = 12, P2 = 35, P3 = 64, P4 = 7. In the seven tied cases within P4, the accommodating style was never among the tied maxima; the accommodating share of P4 (11/55) therefore does not depend on the tie-breaking rule.

Table S8. Full re-estimation bootstrap of the profile × dominant-style distribution (B = 2,000): point estimates, bootstrap means, bias, and 95% percentile confidence intervals (%).

Profile	Style	Point estimate	Bootstrap mean	Bias	95% CI lower	95% CI upper
P1	Competing	14.08	14.5	0.42	4.65	29.41
P1	Problem-solving	9.86	7.95	-1.91	0	17.11
P1	Compromising	19.72	15.55	-4.16	0	28.75
P1	Avoiding	38.03	40.61	2.58	26.36	55.39
P1	Accommodating	18.31	21.38	3.07	8.93	38.46
P2	Competing	6.8	7.3	0.5	3.23	12.39
P2	Problem-solving	10.2	11.35	1.14	5.88	18.47
P2	Compromising	31.29	32.77	1.47	25.27	40.72
P2	Avoiding	36.05	34.29	-1.76	26.05	42.28
P2	Accommodating	15.65	14.3	-1.35	9.09	20.86
P3	Competing	9.94	9.98	0.04	6.14	13.98
P3	Problem-solving	23.4	22.8	-0.6	17.22	28.57
P3	Compromising	33.01	32.68	-0.33	26.76	38.63
P3	Avoiding	24.36	24.89	0.53	19.37	30.77
P3	Accommodating	9.29	9.66	0.36	6.29	13.6

Profile	Style	Point estimate	Bootstrap mean	Bias	95% CI lower	95% CI upper
P4	Competing	7.27	8.46	1.18	2.08	18.1
P4	Problem-solving	20	22.67	2.67	12.16	34.62
P4	Compromising	36.36	34.21	-2.16	22.76	46.16
P4	Avoiding	16.36	17.24	0.87	6	27.18
P4	Accommodating	20	17.42	-2.58	8.11	29.55

Table S9. Bootstrap confidence intervals for the key P4 quantities (B = 2,000; percentage points).

Quantity	Point estimate	Bootstrap mean	Bias	95% CI lower	95% CI upper	CI includes 0
P4 problem-solving %	20	22.67	2.67	12.16	34.62	no
P4 accommodating %	20	17.42	-2.58	8.11	29.55	no
P4 − P3, accommodating	10.71	7.77	-2.94	-2.62	20.85	yes
P4 − P1, problem-solving	10.14	14.72	4.58	1.47	29.14	no
P4 − P2, problem-solving	9.8	11.33	1.53	-1.26	24.66	yes
P3 − P4, problem-solving	3.4	0.13	-3.27	-13.73	12.66	yes

Note. The P4 − P3 accommodating difference is not stable under resampling (95% CI includes zero), whereas the P4 − P1 problem-solving difference is. The P3 − P4 problem-solving difference is centered on zero, indicating no P3 peak relative to P4.

Table S10. Leave-one-out jackknife of the P4 dominant-style distribution (55 deletions; fixed profile membership; %).

Style	Full P4 (%)	Minimum	Maximum	SD (pp)
Competing	7.27	5.56	7.41	0.485
Problem-solving	20	18.52	20.37	0.748
Compromising	36.36	35.19	37.04	0.899
Avoiding	16.36	14.81	16.67	0.691
Accommodating	20	18.52	20.37	0.748

Note. No single deletion reverses the P4 > P3 accommodating relation at the dominant-style level (P3 = 9.29%).

Table S11. Tie-breaking sensitivity: association between profile and dominant style under three treatments of tied cases.

Variant	N	$\chi^2(12)$	p	Cramér's V
(a) Original column order	585	37.24	<.001	0.146
(b) Tie cases excluded	467	32.99	<.001	0.153
(c) Random resolution, mean of 1,000	585	34.74	0.0013	0.14
(c) 2.5th percentile	585	27.39		0.125
(c) 97.5th percentile	585	43		0.157

Note. Random resolution: 1,000 iterations drawing uniformly among tied modes; percentile rows give the 2.5th and 97.5th percentiles of the χ^2 and V distributions (each computed column-wise). The association remained significant ($p < .05$) in all 1,000 iterations.

Table S12. Status of four focal claims across the 1,000 random tie resolutions.

Claim	Iterations holding (of 1,000)
P4 problem-solving > P1 and P2	1000
P4 accommodating > P3 accommodating	1000
P3 accommodating lowest of all profiles	1000
P4 accommodating highest of all profiles	45

Table S13. Change in the accommodating share by profile from the original coding (a) to random tie resolution (c); %.

Profile	Original coding	Random resolution (mean)	Change (pp)
P1	18.31	21.61	3.3
P2	15.65	21.42	5.78

Profile	Original coding	Random resolution (mean)	Change (pp)
P3	9.29	13.86	4.56
P4	20	20	0

Note. The original column order deflated the accommodating share in P1–P3 but not in P4 (whose ties never involved accommodating). Under fair resolution, the P1 and P2 shares rise above the P4 share, which is why the claim that P4 has the highest accommodating share holds in only 45 of 1,000 iterations.

S4. Compositional Analysis of the Conflict-Mode Profile: Detailed Tables

The TKI yields ipsative scores with a fixed row sum (30). 43 cases with row sums differing from 30 were excluded, leaving N = 542 (P1 = 66, P2 = 138, P3 = 289, P4 = 49). Zeros (competing: 68 cases; accommodating: 2) were handled by multiplicative replacement ($\delta = 0.5$); the composition was then closed and transformed to centered log-ratios (CLR). Profile membership was fixed from the primary LPA solution.

Table S14. CLR means of the five conflict modes by profile, with 95% confidence intervals.

Profile	Mode	CLR mean	95% CI lower	95% CI upper
P1	Competing	-0.69	-0.902	-0.479
P1	Problem-solving	0.002	-0.089	0.093
P1	Compromising	0.183	0.097	0.269
P1	Avoiding	0.352	0.261	0.444
P1	Accommodating	0.153	0.011	0.296
P2	Competing	-0.91	-1.046	-0.775
P2	Problem-solving	0.055	-0.012	0.123
P2	Compromising	0.294	0.239	0.349
P2	Avoiding	0.364	0.307	0.422
P2	Accommodating	0.197	0.114	0.279
P3	Competing	-0.628	-0.723	-0.533
P3	Problem-solving	0.156	0.112	0.2
P3	Compromising	0.292	0.253	0.331
P3	Avoiding	0.22	0.176	0.265
P3	Accommodating	-0.041	-0.11	0.028
P4	Competing	-0.434	-0.601	-0.268
P4	Problem-solving	0.168	0.08	0.255
P4	Compromising	0.243	0.154	0.332
P4	Avoiding	0.098	0.007	0.189
P4	Accommodating	-0.075	-0.226	0.076

Table S15. One-way ANOVAs of the CLR-transformed modes across profiles, with Holm-adjusted p values.

Mode	F(3, 538)	p	η^2	Holm-adjusted p
Competing	5.71	< .001	0.031	0.0022
Problem-solving	4.58	0.0035	0.025	0.0071
Compromising	2.23	0.0833	0.012	0.0833
Avoiding	9.49	< .001	0.05	< .001
Accommodating	7.15	< .001	0.038	< .001

Table S16. All pairwise profile contrasts on the CLR-transformed modes (Tukey HSD).

Mode	Contrast	Δ (mean difference)	95% CI	p (Tukey)	d
Competing	P1–P2	-0.22	[-0.529; 0.089]	0.259	-0.27
Competing	P1–P3	0.063	[-0.219; 0.345]	0.94	0.08
Competing	P1–P4	0.256	[-0.134; 0.646]	0.329	0.32
Competing	P2–P3	0.283	[0.069; 0.497]	0.004	0.35

Mode	Contrast	Δ (mean difference)	95% CI	p (Tukey)	d
Competing	P2–P4	0.476	[0.132; 0.820]	0.002	0.59
Competing	P3–P4	0.193	[-0.126; 0.513]	0.403	0.24
Problem-solving	P1–P2	0.054	[-0.092; 0.199]	0.777	0.14
Problem-solving	P1–P3	0.154	[0.022; 0.287]	0.015	0.41
Problem-solving	P1–P4	0.166	[-0.018; 0.349]	0.093	0.44
Problem-solving	P2–P3	0.101	[0.000; 0.201]	0.05	0.27
Problem-solving	P2–P4	0.112	[-0.050; 0.274]	0.282	0.3
Problem-solving	P3–P4	0.011	[-0.139; 0.162]	0.997	0.03
Compromising	P1–P2	0.111	[-0.018; 0.239]	0.118	0.33
Compromising	P1–P3	0.109	[-0.008; 0.226]	0.077	0.33
Compromising	P1–P4	0.06	[-0.101; 0.222]	0.771	0.18
Compromising	P2–P3	-0.002	[-0.090; 0.087]	1	0
Compromising	P2–P4	-0.05	[-0.193; 0.092]	0.799	-0.15
Compromising	P3–P4	-0.049	[-0.181; 0.084]	0.778	-0.15
Avoiding	P1–P2	0.012	[-0.129; 0.153]	0.996	0.03
Avoiding	P1–P3	-0.132	[-0.260; -0.004]	0.041	-0.36
Avoiding	P1–P4	-0.254	[-0.432; -0.077]	0.001	-0.7
Avoiding	P2–P3	-0.144	[-0.241; -0.047]	0.001	-0.39
Avoiding	P2–P4	-0.266	[-0.423; -0.110]	0	-0.73
Avoiding	P3–P4	-0.122	[-0.268; 0.023]	0.135	-0.33
Accommodating	P1–P2	0.043	[-0.173; 0.260]	0.955	0.08
Accommodating	P1–P3	-0.194	[-0.392; 0.003]	0.056	-0.35
Accommodating	P1–P4	-0.228	[-0.501; 0.045]	0.138	-0.41
Accommodating	P2–P3	-0.238	[-0.388; -0.088]	0	-0.42
Accommodating	P2–P4	-0.272	[-0.512; -0.031]	0.02	-0.48
Accommodating	P3–P4	-0.034	[-0.258; 0.190]	0.98	-0.06

Note. d = mean difference divided by the root mean square error of the corresponding ANOVA. Positive Δ indicates a higher value in the second-listed profile.

Table S17. Orthogonalized linear and quadratic trend tests of the CLR-transformed modes across profiles (profiles coded 1–4).

Mode	Linear b	p (linear)	R^2 (linear)	Quadratic b	p (quadratic)	ΔR^2 (quadratic)
Competing	0.1564	0.000634	0.0144	0.0965	0.025719	0.0091
Problem-solving	0.0653	0.002335	0.0225	-0.0122	0.547668	0.0007
Compromising	0.007	0.708067	0.0029	-0.0392	0.028478	0.0088
Avoiding	-0.1065	0	0.0425	-0.0315	0.10888	0.0046
Accommodating	-0.1222	0.000145	0.0279	-0.0137	0.651423	0.0004

Table S18. Omnibus test and Aitchison distances between profile centroids.

MANOVA on four isometric log-ratio coordinates: Wilks' $\Lambda = .9048$, $F(12, 1415,8) = 4.55$, $p = 3.0e\text{-}07$, partial $\eta^2 = .033$.

	P1	P2	P3	P4
P1	0	.256	.308	.462
P2	.256	0	.409	.622
P3	.308	.409	0	.237
P4	.462	.622	.237	0

Note. The closest pair of centroids is P3–P4 (0.237) and the most distant is P2–P4 (0.622).

S5. Subgroup Congruence of the Profile Structure

Table S19. Four-profile solutions estimated separately by gender, and their congruence with the full-sample profiles.

Subsample	n	Profile sizes (P1/P2/P3/P4)	Entropy	φ P1	φ P2	φ P3	φ P4	Mean φ
Women	460	41/151/213/55	0.8	0.984	0.983	0.998	0.997	0.99
Men	85	14/30/30/11	0.846	0.993	0.962	0.964	0.994	0.978

Note. φ = Tucker's congruence between the subsample and full-sample profile mean vectors, matched by elevation order. The female-subsample solution reproduces the full-sample structure almost exactly (all φ ≥ .98). The male subsample is reported descriptively only: with n = 85 and 43 free parameters, the estimates are near the feasibility limit of the model and preclude firm conclusions.

S6. Reproducibility

Software: Python 3.12; StepMix 3.0.0; NumPy/SciPy; statsmodels 0.14.6. Seeds: primary LPA random_state = 42; bootstrap resampling generator seed = 42 with per-replication StepMix seeds 1000 + b; BLRT data-generation seeds 50000 + 1000·c + b with estimation seeds 90000/91000 + 1000·c + b; random tie resolution generator seed = 42. Within-bootstrap estimation used 10 random initializations, a setting validated in a pilot in which it recovered the 20-initialization solution in 20 of 20 bootstrap samples.
